# High-density lipoprotein cholesterol efflux capacity is inversely associated with cardiovascular risk: a systematic review and meta-analysis

**DOI:** 10.1186/s12944-017-0604-5

**Published:** 2017-11-10

**Authors:** Chengfeng Qiu, Xiang Zhao, Quan Zhou, Zhen Zhang

**Affiliations:** 1 Xiangya school of Pharmaceutical Sciences, Central South University, Changsha, Hunan 410013 China; 2 Center for Vascular Disease and Translational Medicine, The Third Xiangya Hospital of Central South University, Changsha, Hunan 410013 China; 3 Department of Pharmacy, The First People’s Hospital of Huaihua City, Huaihua, 418000 China; 4 Department of Emergency, The First People’s Hospital of Huaihua City, Huaihua, Hunan 418000 China; 5 Department of Science and Education, The First People’s Hospital of Changde City, Changde, Hunan 415003 China; 6 Centre for Experimental Medicine, Third Xiangya Hospital of Central South University, Changsha, Hunan 410013 China

**Keywords:** High-density lipoprotein, Cholesterol efflux capacity, Cardiovascular risk, Meta-analysis

## Abstract

**Background:**

A low plasma level of high-density lipoprotein (HDL) cholesterol (HDL-C) is associated with cardiovascular risk. A key cardioprotective property of HDL is cholesterol efflux capacity (CEC), the ability of HDL to accept cholesterol from macrophages. In this study, we aimed to identify the predictive value of CEC for cardiovascular risk.

**Methods:**

The relative risks (RRs) and 95% confidence intervals (CIs) were pooled to analyze the association between CEC and the incidence of cardiovascular events and all-cause mortality. The odds ratios (ORs) and 95% CIs were pooled to estimate the association of CEC and the prevalence of cardiovascular events.

**Results:**

A total of 15 studies were included. Results showed that the highest CEC was significantly associated with a reduced risk of cardiovascular events incidents compared to the lowest CEC (RR, 0.56; 95% CI, 0.37 to 0.85; *I* ^2^, 89%); the pooled RR of cardiovascular risk for per unit SD increase was 0.87 (95% CI, 0.73 to 1.04; *I* ^2^, 67%). Dose-response curve indicated that cardiovascular risk decreased by 39% (RR, 0.61; 95% CI, 0.51 to 0.74) for per unit CEC increase. Similarly, an inverse association was observed between CEC and the prevalence of cardiovascular events (highest vs. lowest, OR, 0.30; 95% CI, 0.17 to 0.5; *I* ^2^ = 63%; per unit SD increase, OR, 0.94; 95% CI, 0.90 to 0.98; *I* ^2^ = 71%). However, based on the current data, CEC was not significantly associated with all-cause mortality.

**Conclusions:**

Findings from this meta-analysis suggest that HDL-mediated CEC is inversely associated with cardiovascular risk, which appears to be independent of HDL concentration. The growing understanding of CEC and its role in cardiovascular risk decrease may improve the accuracy of cardiovascular risk prediction and also open important avenues to develop novel therapeutic targeting HDL metabolism.

**Electronic supplementary material:**

The online version of this article (doi: 10.1186/s12944-017-0604-5) contains supplementary material, which is available to authorized users.

## Background

High-density lipoprotein (HDL) cholesterol (HDL-C) is ubiquitously regarded as the “good cholesterol”, and its complex relationship with cardiovascular risk has been a topic of great interest for decades. Epidemiologic studies revealed a robust and inverse association between low HDL-C and cardiovascular risk [1]. This association gave rise to the hypothesis that “higher HDL is better”. However, the hypothesis has been challenged recently on the basis of clinical and genetic studies. Pharmacological interventions such as niacin and cholesteryl ester transfer protein (CETP) inhibitors, increased HDL-C but failed to reduce cardiovascular risk [2–5]. Genetic studies in humans also revealed that certain genetic variants associated with increased HDL-C do not protect the carriers against cardiovascular disease (CVD) [6]. HDL particles vary in lipid and protein composition and HDL subspecies serve different biological functions [7]; thus, HDL concentration does not accurately reflect the biological function. This suggests the limited value of using steady-state HDL concentrations to assess the cardioprotective effects of HDL.

HDLs exert several activities to provide protection against the development of atherosclerosis; these functions are related to reverse cholesterol transport (RCT) as well as anti-inflammatory, endothelial and vascular functions [8]. Among them, RCT is considered to be the key cardioprotective property of HDL. RCT is the complex process by which HDLs accept cholesterol derived from artery-wall macrophages and mediate delivery to the liver for disposition [8, 9]. Macrophages laden with excessive cholesteryl esters form foam cells, which are implicated in the causal pathway of atherosclerosis [10, 11]. By functioning as an acceptor of cholesterol derived from macrophages, HDLs can inhibit and/or reduce foam cell formation. Cholesterol efflux from macrophages is the initial step of RCT and the cholesterol efflux capacity (CEC) is commonly used to assess the biological function of HDLs in ex vivo [7].

In recent years, ex vivo CEC assays have been successfully used in clinical studies to measure the capacity of apolipoprotein B (apoB)-depleted serum to accept labeled cholesterol from macrophage cells [12]. Although recent studies have been conducted to investigate the association between CEC and cardiovascular risk, the results of these studies are inconsistent.

Accordingly, in this study, we performed a standard meta-analysis and dose-response analysis to estimate the association between CEC and cardiovascular risk to identify the predictive value of CEC for cardiovascular risk. This information is important in guiding the development of the HDL-targeted therapies that reduce cardiovascular risk.

## Methods

This study was performed according to the guidelines proposed by the Meta-analysis of Observational Studies in Epidemiology (MOOSE) group. The Research plan was defined prior to conducting the review.

### Date sources and extraction

Studies of the relationship between HDL-mediated CEC and cardiovascular risk were considered to be eligible for inclusion. Two investigators independently searched for the relevant studies in the MEDLINE, EMBASE and Cochrane Library databases. Literature searches were last updated on February 1st, 2017. The following groups of key words were used in literature searches: “cholesterol efflux capacity” OR “HDL-mediated cholesterol efflux”, and “cardiovascular events” OR “mortality” OR “all-cause death” OR “cardiovascular risk” OR “acute coronary syndromes” OR “myocardial infarction” OR “cardiovascular disease” OR “risk” OR “death” OR “mortality” OR “outcome” OR “stroke” OR “transient ischemic attacks” OR “intracranial hemorrhage” OR “events”. An additional manual search of the reference lists of original articles and review articles was also performed.

Two investigators independently conducted the study selection on the basis of predefined criteria by reviewing the abstract and full-text. Studies that reported the association between HDL-mediated CEC and human cardiovascular risk were included in the initial study selection process. Outcomes of included studies were identified as the incidence and prevalence of cardiovascular events, and all-cause mortality. Cardiovascular events were defined as a composite of atherosclerotic cardiovascular events and death from cardiovascular causes. If multiple studies researched the same population, the study with the most detailed data or the larger sample size was selected. Studies were excluded if they failed to meet all the listed inclusion criteria. One investigator extracted data, while a second checked the data for accuracy. All disagreements were resolved in consultation with a third investigator.

The following information was extracted from each of the eligible studies: first author’s name, publication year, country, sample size, clinical characteristics of participants, age, male/female, follow-up time, CEC assay methods, CEC level, events, effect size and corresponding 95% CI, adjusted factors.

### CEC assay methods

Ex vivo CEC assays, which are readily used in clinical studies, consist of three components are involved: labeled cholesterol, a donor cell which releases the labeled cholesterol, and a cholesterol acceptor, which are prepared from clinical serum samples using standard methods. To reduce the variation between studies, three elements of CEC assays were identified as follows: (i) donor cell lines were macrophages (J774 cells or THP-1 cells); (ii) cholesterol was radiolabeled with ^3^H or labeled with fluorescence; (iii) and serum samples from clinical subjects which were processed to remove apoB were used as cholesterol acceptors.

### Quality assessment

Two investigators independently assessed the quality of each study by applying the criteria defined by the Newcastle–Ottawa Scale (NOS) score, which is widely used in the assessment of the quality of observational studies. Three factors comprising selection, comparability and outcome are used in the assessment criteria, and the scores range from 0 to 9.

### Data synthesis and analysis

In the current meta-analysis, we used relative risk (RR) with corresponding 95% confidence interval (CI) as a measure of the effect size to evaluate the association between HDL-mediated CEC and the incidence of cardiovascular events and all-cause mortality. The effect size was calculated based on comparison of the highest versus the lowest CEC, plus 1 SD CEC increase. Multivariable-adjusted RRs or hazard ratios (HRs) of the original studies were pooled. The odds ratios (ORs) and 95% CIs were pooled to estimate the association between CEC and the prevalence of cardiovascular events. For the dose-response meta-analysis, we used the “generalized least squares for trend estimation” method proposed by Greenland and Longnecker [13, 14] to take into account the correlation with the log RR estimates across the levels of CEC. This method requires knowledge of the cases and cohort size/control subjects of each category and the RR with its variance estimate for at least three quantitative exposure categories. The value assigned to each level of cholesterol efflux capacity was the median/mean provided by original research. We estimated the potential dose-response relationship in two-stages. In the first stage, we estimated a restricted cubic spline model with three knots at percentiles 10, 50 and 90% of the distribution of levels of cholesterol efflux capacity. In the second stage, the two regression coefficients (3 knots minus 1) and the variance/covariance matrix within each study were combined in a multivariate random-effects meta-analysis. A *P*-value for nonlinearity was calculated by testing the null hypothesis that the coefficient of the second spline is equal to 0.

Between-study heterogeneity was assessed by the chi-squared-based Q test and *I* ^2^ statistics [15]. A *P-*value <0.10 for the Q test and *I* ^2^ > 50 were considered to indicate significant heterogeneity. Publication bias was assessed by visual inspection of funnel plots for asymmetry and statistical evaluation with Begg’s rank correlation test [16] and Egger’s linear regression test [17]. Two-tailed α level of significance was set at 0.05.

Statistical analyses were performed with STATA/SE.12.0 (StataCorp, College station, Texas, USA), Review Manager Version 5.3 (The Nordic Cochrane Center, Copenhagen, Denmark) and R version 3.2.0 (R Foundation for Statistical Computing, Vienna, Austria).

## Results

### Baseline characteristics and quality of included studies

**Fig. 1:**
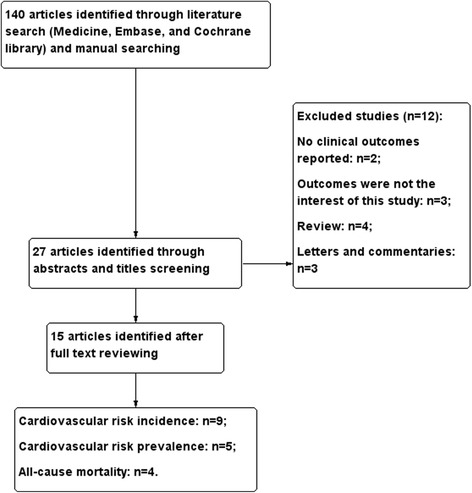
Flow diagram of study selection process

Figure 1 shows the study selection process. A total of 140 articles were considered to be potentially relevant studies after comprehensive searches of the PubMed, EMBASE and Cochrane library databases. A total of 15 articles with 16,364 participants identified by full-text reviews were included in the final meta-analysis. The ages of participants ranged from 42 to 72 years, and 55% (9027) were males. We included 12 cohort studies from nine published articles (*n* = 13,754) to evaluate the association between CEC and the incidence of cardiovascular events; the median follow-up was 5.6 years. In addition, four studies reporting the multivariable-adjusted HR or RR for the incidence of cardiovascular events with different categories of CEC, were included in the dose-response analysis. Eight studies from five articles (*n* = 4732) were included to evaluate the prevalence of cardiovascular events and five studies from four articles (*n* = 3940) were included to assess the all-cause mortality risk. The quality of the included studies was assessed by NOS; scores ranged from 5 to 9 (Table 1 and Additional file 1). Full details of the baseline characteristics of the included articles are presented in Table 1. Events and effect sizes (HR or RR or OR) of original studies are listed in Table 2.

**Table 1:** Main characteristics and quality of included studies

Study ID	Country	Study type	Subjects No. (Male, %)	Subject characteristics	Age, y	Follow-up, y	Donor cell line	Labeled-cholesterol	Cholesterol acceptor	NOS
Bauer, L, 2017 [30]	Germany	Cohort	526 (59)	CKD	65 ± 12	4.6	J774	^3^H–cholesterol	ApoB-depleted plasma	7
Tejera–Segura, B 2017 [31]	Spanish	Cross-sectional	401 (26)	RA	57 ± 10	/	J774	BODIPY-cholesterol	ApoB-depleted plasma	5
Kopecky, C, 2016 [32]	German	Cohort	1147 (54.8)	Hemodialysis patients	66	4.1	THP-1	^3^H–cholesterol	ApoB-depleted plasma	7
Javaheri, A, 2016 [33]	USA	Cohort	35 (85.7)	Cardiac transplant recipients	44 ± 4	2.6	J774	^3^H–cholesterol	ApoB-depleted plasma	6
Mody, P, 2016 [34]	USA	Cohort	1972 (44)	Free from CVD	45 ± 9	9.4	J774	BODIPY-cholesterol	ApoB-depleted plasma	8
Liu, C 2016 [35]	China	Cohort	1737 (65.2)	CAD	64	3.8	J774	BODIPY-cholesterol	ApoB-depleted plasma	6
Zhang, J 2016 [36]	China	Cohort	313 (75.4)	CAD	66 ± 11	3	J774	^3^H–cholesterol	ApoB-depleted plasma	6
Ogura, M, 2016 [37]	Japan	Cross-sectional	227 (44.5)	Heterozygous FH	57 ± 17	/	J774	^3^H–cholesterol	ApoB-depleted plasma	6
Annema, W, 2016 [38]	Netherlands	Cohort	495 (54.5)	Renal Transplant Recipients	52 ± 12	7	THP-1	^3^H–cholesterol	ApoB-depleted plasma	8
Ishikawa, T, 2016 [39]	Japan	Case-control	258 (76.7)	CAD VS non-CAD	66 ± 11	/	J774	^3^H–cholesterol	ApoB-depleted plasma	5
Saleheen, D, 2015 [40]	UK	Nested case-control	3494 (64.5)	CHD vs. non-CHD	65 ± 8;	6	J774	^3^H–cholesterol	ApoB-depleted plasma	8
Rohatgi, A, 2014 [12]	Dallas	Cohort	2924 (43)	Free from CVD	42	9.4	J774	BODIPY-cholestero	ApoB-depleted plasma	9
Li, X M, 2013 [41]	USA	Case-control	1150 (63.8)	CAD vs. non-CAD	64 ± 11	/	J774	^3^H–cholesterol	ApoB-depleted plasma	6
Khera, A V1, 2011 [29]	Caucasian	Cross-sectional	793 (59.7)	CAD vs. non-CAD	58 ± 10	/	J774	^3^H–cholesterol	ApoB-depleted plasma	6
Khera, A V, 2011 [29]	Caucasian	Cross-sectional	203 (54.2)	Healthy population	51 ± 8	/	J774	^3^H–cholesterol	ApoB-depleted plasma	6

Notes: *NOS* Newcastle-Ottawa Scale, *CKD* chronic kidney disease, *RA* rheumatoid arthritis, *DM* diabetes mellitus, *ESR* erythrocyte sedimentation rate, *CVD* cardiovascular disease, *CAD* coronary artery disease, *CHD* coronary heart disease events, *FH* familial hypercholesterolemia, *ApoB* apolipoprotein B

**Table 2:** Effect sizes of included studies in this meta-analysis

Study ID	CEC (median/mean)	Events	HR/RR/OR (95% CI)	Adjustment factors
Bauer, L 2017 [30]	12.2 ± 2.4	CVE	Q1: 1; Q2: 0.58 (0.33–1.00); Q3: 0.67 (0.39–1.15); Q4: 0.91 (0.51–1.62);	Age, sex, BMI, BP, smoking status, GFR, and log albuminuria
Tejera-Segura, B 2017 [31]	Control: 16.9 ± 10.4; Case: 18.9 ± 9.0	Subclinical atherosclerosis	0.94 (0.89–0.98)	Age, sex, SBP, DM, ESR, DAS28 and tocilizumab use
Kopecky, C, 2016 [32]	T1: 0.73; T2: 0.89; T3: 1.08	CVD	0.92 (0.83–1.02)	Traditional risk factors, LDL-C, HDL-C, apoA-I, and CRP
Javaheri, A, 2016 [33]	Alive: 0.98 ± 0.03; Dead: 0.89 ± 0.03.	Mortality	0.19 (0.06–0.56)	HDL-C, LDL-C, ischemic origin, and rejection
Mody, P, 2016 [34]	/	CVD	0.35 (0.23–0.55)	TC, HDL-C, history of blood pressure medication use, BMI, and CRP
Liu, C 2016 [35]	Q1: 0.70 (0.17–0.79); Q2: 0.86 (0.79–0.93); Q3: 1.00 (0.93–1.07); Q4: 1.15 (1.07–2.01)	All-cause death and cardiovascular death	All-cause death: 0.10 (0.01–0.74); Cardiovascular death: 0.08 (0.01–0.68)	Age, sex, BMI, smoking and alcohol drinking, hypertension, DM, dyslipidemia, lipid-lowering drug use, TC, TG, LDL-C, HDL-C, and apoA-I
Zhang, J 2016 [36]	/	CVD	0.30 (0.14–0.67)	Age, sex, hypertension, diabetes, current smoking, LDL-C, HDL-C, Apo A, Apo B, and regular medication
Ogura, M, 2016 [37]	/	Incidence of CVD	0.95 (0.90–0.99)	Age, sex, hypertension, diabetes mellitus, smoking history, obesity, LDL-C, TG, HDL-C
Annema, W, 2016 [38]	T1 (%): 5.8(5.3–6.4); T2 (%): 7.3(6.8–7.9); T3 (%): 9.0(8.2–9.8)	All-cause death and cardiovascular death	CV mortality: 0.96 (0.72–1.27); All-cause mortality: 0.84 (0.68–1.04)	Age, sex, apo A-I, HDL-C cholesterol and creatinine clearance
Ishikawa, T, 2016 [39]	CAD: 0.86 ± 0.26; Non-CAD: 1.02 ± 0.38	Incidence of CVD	0.23 (0.056–0.91)	Baseline adjustment
Saleheen, D, 2015 [40]	/	Incidence of CHD events	Top vs. bottom: 0.64 (0.51–0.80); Per 1 SD: 0.80 (0.70–0.90)	Age, sex, diabetes, hypertension, cigarette use, alcohol use, waist:hip ratio and BMI, LDL-C, TG and HDL-C
Rohatgi, A, 2014 [12]	0.21–3.93	Incidence of CVD	0.33 (0.109–0.55)	Age, sex, race, diabetes, hypertension,smoking, BMI, TG, TC, and statin use
Li, X M, 2013 [41]	/	Incidence of CAD and MACE	1.85 (1.11–3.06)	Age, sex, smoking, diabetes mellitus, hypertension, LDL-C, and HDL-C
Khera, A V, 2011 [29]	Case: 0.82; Control: 0.9	CAD	Per 1 SD increase: 0.75 (0.63–0.90); Q4 vs. Q1: 0.48 (0.30–0.78)	Cardiovascular risk factors and HDL-C
Khera, A V, 2011 [29]	0.77(0.36–1.68)	CVD prevalence	Per 1 SD increase: 0.97 (0.94–0.99)	Age, sex, cardiovascular risk factors a nd HDL-C

Notes: *CEC* cholesterol efflux capacity, *RR* risk ratio, *HR* hazard ratio, *CI* confidence interval, *OR* odds ratio, *NOS* Newcastle-Ottawa Scale, *CKD* chronic kidney disease, *CVE* cardiovascular event, *BMI* body mass index, *BP* blood pressure, *SBP* systolic blood pressure, *GFR* glomerular filtration rate, *RA* rheumatoid arthritis, *DM* diabetes mellitus, *ESR* erythrocyte sedimentation rate, *CVD* cardiovascular disease, *LDL-C* low density lipoprotein-cholesterol, *HDL-C* high-density lipoprotein-cholesterol, *Apo* apolipoprotein, *CRP* C-reactive Protein, *TC* total cholesterol, *CAD* coronary artery disease, *Q* quartile, *TG* triglyceride, *CHD* coronary heart disease events, *T* tertiles, *MACE* major adverse cardiovascular event

### The association between HDL-mediated CEC and the incidence of cardiovascular events

**Fig. 2:**
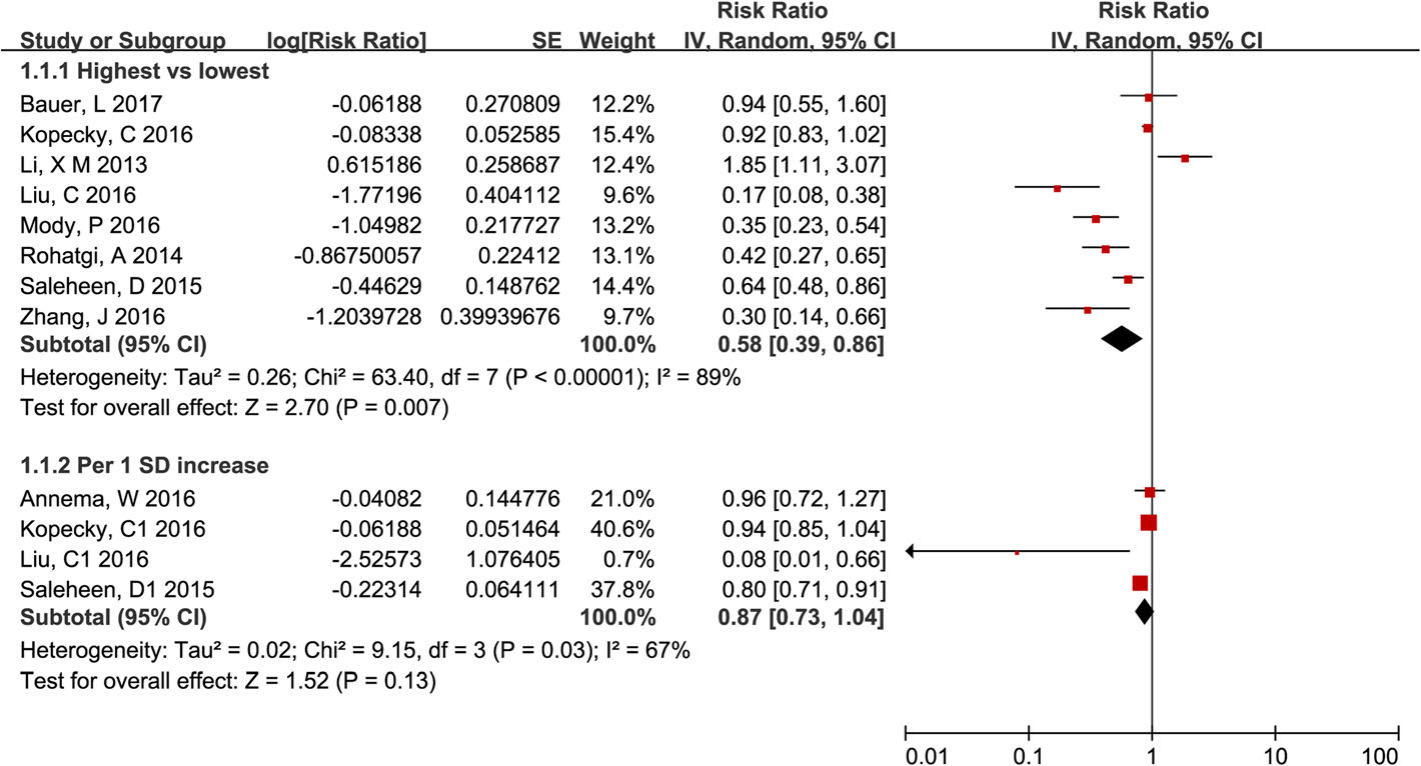
Pooled estimates of the relative risk of the incidence of cardiovascular events associated with CEC

When compared to the lowest CEC, the highest levels of CEC were significantly associated with reduced cardiovascular risk (13,259 participants across 8 studies; pooled RR, 0.56; 95% CI, 0.37 to 0.85; *I* ^2^, 89%; Fig. 2). No publication bias was found via visual inspection of funnel plots for asymmetry (Additional file 2) and statistical evaluation with Begg’s and Egger’s tests (*P*-value for Begg’s and Egger’s tests were 0.39 and 0.11, respectively). The pooled RR of the incidence of cardiovascular events for 1 SD increase was 0.87 (6869 participants across 4 studies, 95% CI, 0.73 to 1.04; *I* ^2^, 67%; Fig. 2), and no obvious publication bias was observed (Additional file 2; *P*-value for Begg’s and Egger’s tests were 0.308 and 0.388, respectively). Sensitivity analysis was performed to confirm the robustness of these findings by sequential application of the leave-one-out method to investigate the influence of each individual study on the overall risk estimate. The results did not show any significant change in the pooled effect size (Additional files 3 and 4).

**Fig. 3:**
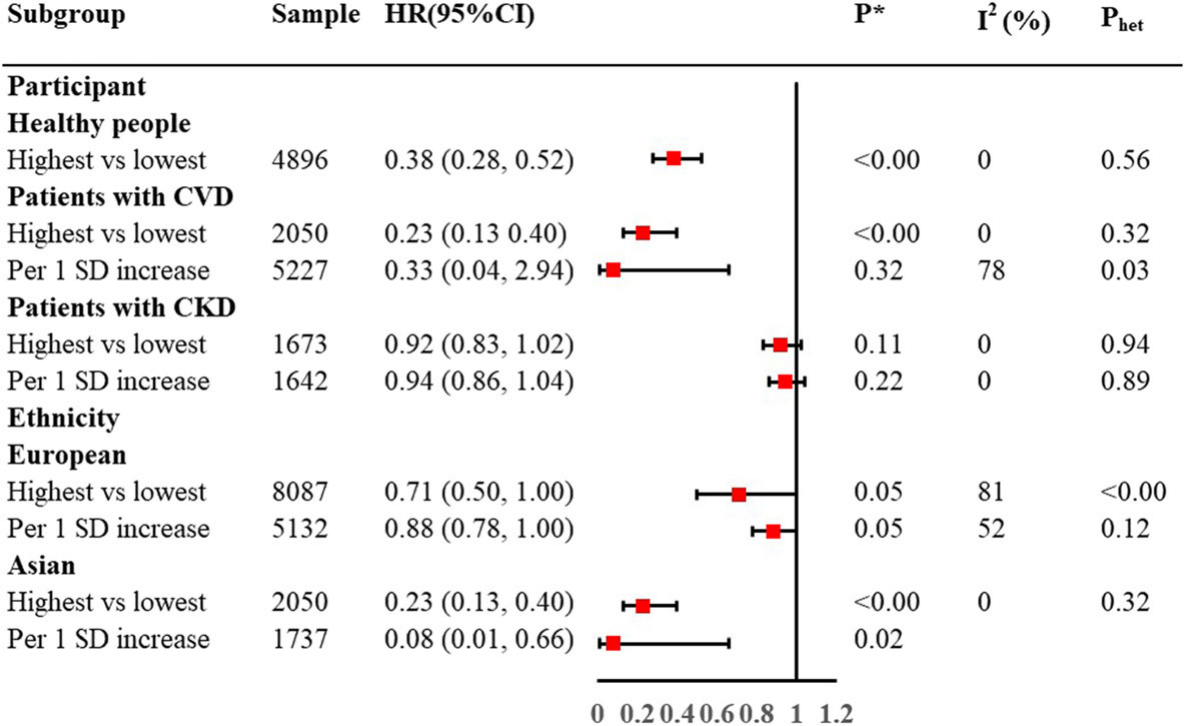
Subgroup analyses of the relative risk of the incidence of cardiovascular events stratified by clinical characteristics of the participant and ethnicity. Notes: P_het_ was utilized to assess the between-study heterogeneity using the chi-squared-based Q test

Subgroup analysis stratified by clinical characteristics of subjects showed a strong association of CEC with the incidence of cardiovascular events in healthy individuals (highest vs. lowest, RR, 0.38; 95% CI, 0.28 to 0.52; *I* ^2^ = 0; Fig. 3) and in patients with baseline CVD (highest vs. lowest, RR, 0.23; 95% CI, 0.13 to 0.40; *I* ^2^ = 0; Fig. 3). However, such an inverse relationship was not demonstrated in the patients with renal impairment (highest vs. lowest, RR, 0.92; 95% CI, 0.83 to 1.02; *I* ^2^ = 0; per 1 SD increase, RR, 0.94; 95% CI, 0.86 to 1.04; *I* ^2^ = 0; Fig. 3). Subgroup analysis stratified by ethnicity demonstrated consistent results among Asian populations (highest vs. lowest, RR, 0.23; 95% CI, 0.13 to 0.40; *I* ^2^ = 0; per 1 SD increase, RR, 0.08; 95% CI, 0.01 to 0.66; *I* ^2^ = 0; Fig. 3), and among European populations (highest vs. lowest, RR, 0.71; 95% CI, 0.50 to 1.00; *I* ^2^ = 81; per 1 SD increase, RR, 0.88; 95% CI, 0.78 to 1.00; *I* ^2^ = 52; Fig. 3).

**Fig. 4:**
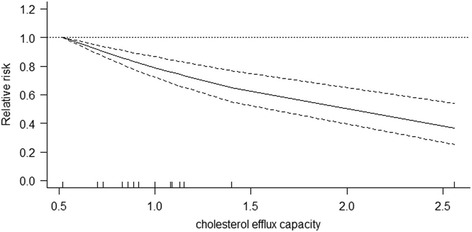
Dose-response association between CEC and cardiovascular risk incidence

Four studies provided data for the dose-response meta-analysis. Using a restricted cubic spline model, we observed a linear dose-response association between CEC and cardiovascular risk [χ^2^ test for non-linearity = 2.2, (df = 2), *P* = 0.33]. The dose-response curve (Fig. 4) indicated that the cardiovascular risk decreased by 39% (RR, 0.61; 95% CI, 0.51 to 0.74) per unit increment in cholesterol efflux capacity.

### The association between HDL-mediated CEC and the prevalence of cardiovascular events

**Fig. 5:**
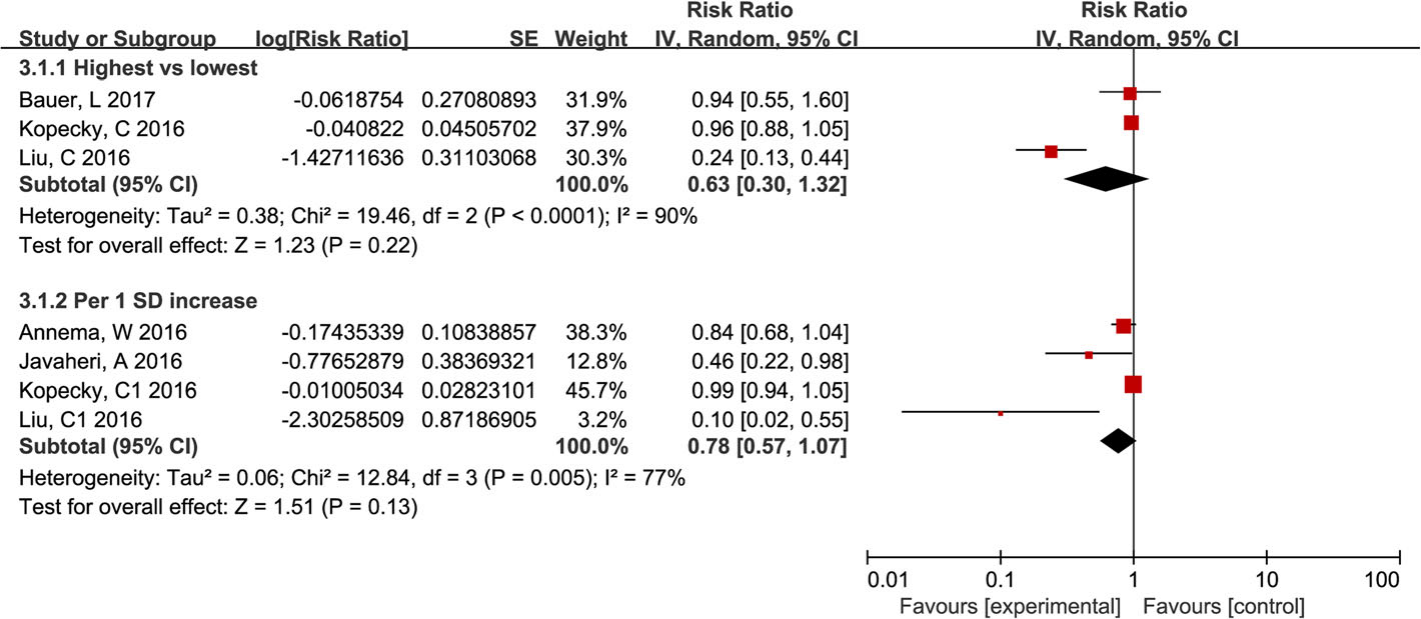
Pooled estimates of the odds ratios of the prevalence of cardiovascular events associated with CEC

Compared with the lowest CEC, the prevalence of cardiovascular events was significantly reduced in the participants with the highest CEC (OR, 0.30; 95% CI, 0.17 to 0.51; *I* ^2^ = 63%; Fig. 3). Pooled results showed a slight reduction in the prevalence of cardiovascular events with per 1 SD increase (OR, 0.94; 95% CI, 0.90 to 0.98; *I* ^2^ = 71%; Fig. 5). Potential publication bias was assessed by visual inspection of funnel plots for asymmetry (Additional file 5) and through an Egger’s linear regression test with a *P*-value of 0.005. Results from sensitivity analysis did not show a significant change (Additional files 6 and 7).

### The association between HDL-mediated CEC and all-cause mortality

**Fig. 6:**
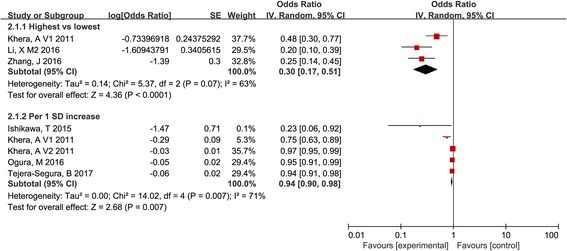
Pooled estimates of the relative risk of all-cause mortality associated with CEC

In the final meta-analysis, a total of 6824 participants were included to evaluate the association between CEC and all-cause mortality. There was no statistically significant difference in the pooled effect size in the comparisons of the highest with lowest CEC, as well as in the comparisons of the data per 1 SD increase (Fig. 6). Visual inspection of funnel plots for asymmetry indicated the potential existence of publication bias (Additional file 8).

## Discussion

To the best of our knowledge, the is the first comprehensive evaluation of the association between HDL-mediated CEC and cardiovascular risk performed by meta-analysis. Three important results were found in this study. First, there was a strong inverse association between HDL-meditated CEC and the incidence of cardiovascular events, especially in healthy individuals and patients with CVD at baseline. Dose-response analysis demonstrated a linear, dose-dependent association, while the cardiovascular risk decreased as the increased CEC. Second, CEC was also inversely associated with the prevalence of cardiovascular events. Third, although there was a trend toward an inverse association between HDL-CEC and all-cause mortality, this was not found to be statistically significant based on the current data.

HDLs comprise various subspecies and vary widely in particle size, as well as the lipid and protein composition [18]. Indeed, it is now widely known that specific HDL subspecies exert highly specific functions [9, 19]. Static concentration measurements are poorly reflecting the biologic activities of HDL [20]. This may account for the failure of pharmacologic interventions (niacin and CETP inhibitors) designed to raise plasma HDL-cholesterol levels to improve cardiovascular outcomes. HDL-mediated cholesterol efflux from macrophages, which is the key functional property of HDL in protecting against atherosclerosis, is a critical process that is evaluated by RCT [7, 21, 22]. In recent years, a growing number of studies have focused on HDL function, especially CEC, instead of simply assessing HDL concentrations, with the aim of gaining an improved understanding of the clinical benefits of HDL in protecting against CVD. However, these clinical studies have yielded inconsistent results, and the link between HDL-mediated CEC and cardiovascular risk remains to be fully clarified.

Importantly, analysis of the pooled results in this study showed that the capacity of HDL to promote cholesterol efflux was inversely associated with the future incidence of cardiovascular events. Furthermore, the dose-response analysis demonstrated that increased CEC was related to decreased cardiovascular risk, in a relationship that is apparently independent of HDL levels. In the subgroup analysis stratified by ethnicity, significant inverse associations between CEC and cardiovascular risk were observed both in European and Asian populations. The pooled effect size (RR) was much smaller in Asian populations than that in European populations. It is well recognized that there are differences in cardiovascular risk between European and Asian populations involving many factors, including genetic factors, lifestyle, and environment. Studies have demonstrated that lifestyle changes also influence CEC [23]. Nevertheless, we were unable to identify ethnicity as a determinant factor that may influence the predictive value of CEC for cardiovascular risk, because of limited data. This is an interesting issue that warrants further investigation. Further subgroup analysis stratified according to the baseline clinical characteristics of participants revealed that the results are influenced by disease. First, we found that HDL-cholesterol efflux capacity was a strong predictor of cardiovascular risk both in healthy individuals and in patients with CVD. As an integrated measure of HDL quantity and quality, CEC accurately reflects the role of HDL in atheroprotection. Studies have demonstrated that HDL-cholesterol efflux exerts multiple cardioprotective properties, the most important being that the cholesterol efflux from macrophages inhibits foam cell formation and protects macrophages from LDL-induced apoptosis [24]. In addition, CEC has been shown to be an important signaling pathway required for nitric oxide activation and protein transport, and enhances endothelial function by mediating anti-inflammatory and anti-oxidant activities [25, 26]. Accordingly, increased CEC enhances the cardioprotective activities of HDL, and vice versa. Although limited data were pooled in this meta-analysis and statistical heterogeneity was present, these results provide important information showing that HDL-mediated macrophage-specific CEC is potentially a stronger predictor of cardiovascular risk among patients with various CVDs and healthy individual. However, CEC was not correlated with cardiovascular risk in the patients with kidney disease. The potential underlying reason for this could be the dysfunction of HDL in kidney disease. HDL particles are characterized by an altered molecular composition under conditions of kidney disease, which is not restricted to the anti-inflammatory and anti-oxidative effects, but also result in decreased CEC [27]. Moreover, these patients usually lose lipoproteins via the urine and as a consequence, it is likely that not only the quality, but also the quantity of HDL is decreased. In fact, cardiovascular risk is notably increased in individuals with chronic kidney disease, mainly due to the high prevalence of traditional risk factors, such as hypertension, diabetes, dyslipidemia and high levels of inflammation [28]. The findings of this study showed that disease characteristics affect the predictive value of CEC for cardiovascular risk. HDL-mediated CEC may be a good predictive biomarker of cardiovascular risk in healthy individuals and in patients with CVD, but not in patients with impaired kidney function.

A previous cross-sectional study reported a strong inverse association between CEC and the prevalence of coronary artery disease [29]. A similar association was found between CEC and the prevalence of cardiovascular risk in this study. Because of the obvious heterogeneity and potential publication bias, the association was weaker and remains to be confirmed by more clinical studies. However, our results did not show a statistical association between CEC and all-cause mortality. This indicates that HDL-mediated CEC is predominantly correlated with cardiovascular risk, but not with the other risks. The small sample (*n* = 6824) included in this meta-analysis and the existence of heterogeneity may reduce the credibility of this result.

The findings of our study may have important clinical implications. Although cholesterol efflux from macrophages represents only a small component of the overall turnover of cholesterol in the body as a whole, it is probably the property that is most relevant to atheroprotection and accurately reflects HDL function. The results of this study suggest a potential role of HDL-mediated CEC in cardiovascular risk prediction and support the use of CEC assays in guiding the development of new HDL-targeted therapies. However, before the measurement of CEC is widely recommended in clinical practice, it is necessary to standardize CEC assays and develop high-throughput platforms that are suitable for routine clinical use.

Several limitations of this study should be recognized. First, combined cardiovascular events were identified as the outcome to estimate the cardiovascular risk in the included studies, thus, making it difficult to identify the risk of specific cardiovascular events, such as myocardial infarctions and ischemic strokes. Larger clinical trials dealing the association of CEC with the incidence of specific cardiovascular events are urgently needed to validate this concept. Second, the pooled results were based on observational studies, and although multiple-adjusted effect size was selected, the adjusted factors were not consistent. Hence, we cannot exclude the impact of the other confounders, which may account for some between-study variation. Finally, the most important limitation is that there is no established gold-standard for ex vivo CEC assays. For example, CEC measurement using different forms of labeled cholesterol (radiolabel or fluorescence label) and differences in donor cell lines (J774 or THP-1) can yield between-study variation. At present, the cholesterol efflux assay is not readily applicable in routine clinical practice. Our findings support the implementation of efforts to establish a standardized CEC assay that is suitable for clinical use.

## Conclusions

In conclusion, the findings of this meta-analysis suggest that HDL-mediated cholesterol efflux capacity is inversely associated with the cardiovascular risk, which appears to be independent of HDL concentrations. A significant linear association revealed that the cardiovascular risk decreased as the CEC increased. The growing understanding of CEC and its role in cardiovascular risk decrease may improve the accuracy of risk prediction and also open important avenues to develop novel therapeutic targeting HDL metabolism.

## Additional files

Additional file 1: Quality assessment of included studies. (XLS 36 kb)

Additional file 2: Funnel plot of CEC and the incidence of cardiovascular events. (TIFF 4376 kb)

Additional file 3: Sensitivity analysis of the association between CEC and the incidence of cardiovascular events (highest vs. lowest CEC). (TIFF 9883 kb)

Additional file 4: Sensitivity analysis of the association between CEC and the incidence of cardiovascular events with 1 SD increase in CEC. (TIFF 9883 kb)

Additional file 5: Funnel plot of CEC and the prevalence of cardiovascular events. (TIFF 7496 kb)

Additional file 6: Sensitivity analysis of the association between CEC and the prevalence of cardiovascular events (highest vs. lowest CEC). (TIFF 6331 kb)

Additional file 7: Sensitivity analysis of the association between CEC and the prevalence of cardiovascular events with 1 SD increase in CEC. (TIFF 6332 kb)

Additional file 8: Funnel plot of CEC and all-cause mortality. (TIFF 4376 kb)

## Abbreviations

Apo: Apolipoprotein
BMI: Body mass index
BP: Blood pressure
CAD: Coronary artery disease
CEC: Cholesterol efflux capacity
CETP: Cholesteryl ester transfer protein
CHD: Coronary heart disease events
CI: Confidence interval
CKD: Chronic kidney disease
CRP: C-Reactive Protein
CVD: Cardiovascular disease
CVE: Cardiovascular event
DM: Diabetes mellitus
ESR: Erythrocyte sedimentation rate
GFR: Glomerular filtration rate
HDL-C: High-density lipoprotein-cholesterol
HR: Hazard ratio
LDL-C: Low density lipoprotein-cholesterol
MACE: Major adverse cardiovascular event
MOOSE: Meta-analysis of Observational Studies in Epidemiology
NOS: Newcastle–Ottawa Scale
OR: Odds ratios
Q: Quartile
RA: Rheumatoid arthritis
RCT: Reverse cholesterol transport
RR: Relative risk
SBP: Systolic blood pressure
T: Tertiles
TC: Total cholesterol
TG: Triglyceride
