# Supplementary figures and images for: High-density lipoprotein cholesterol efflux capacity is inversely associated with cardiovascular risk: a systematic review and meta-analysis
Source: Lipids Health Dis. 2017 Nov 10;16:212. doi: 10.1186/s12944-017-0604-5 (PMC5681808; doi:10.1186/s12944-017-0604-5)

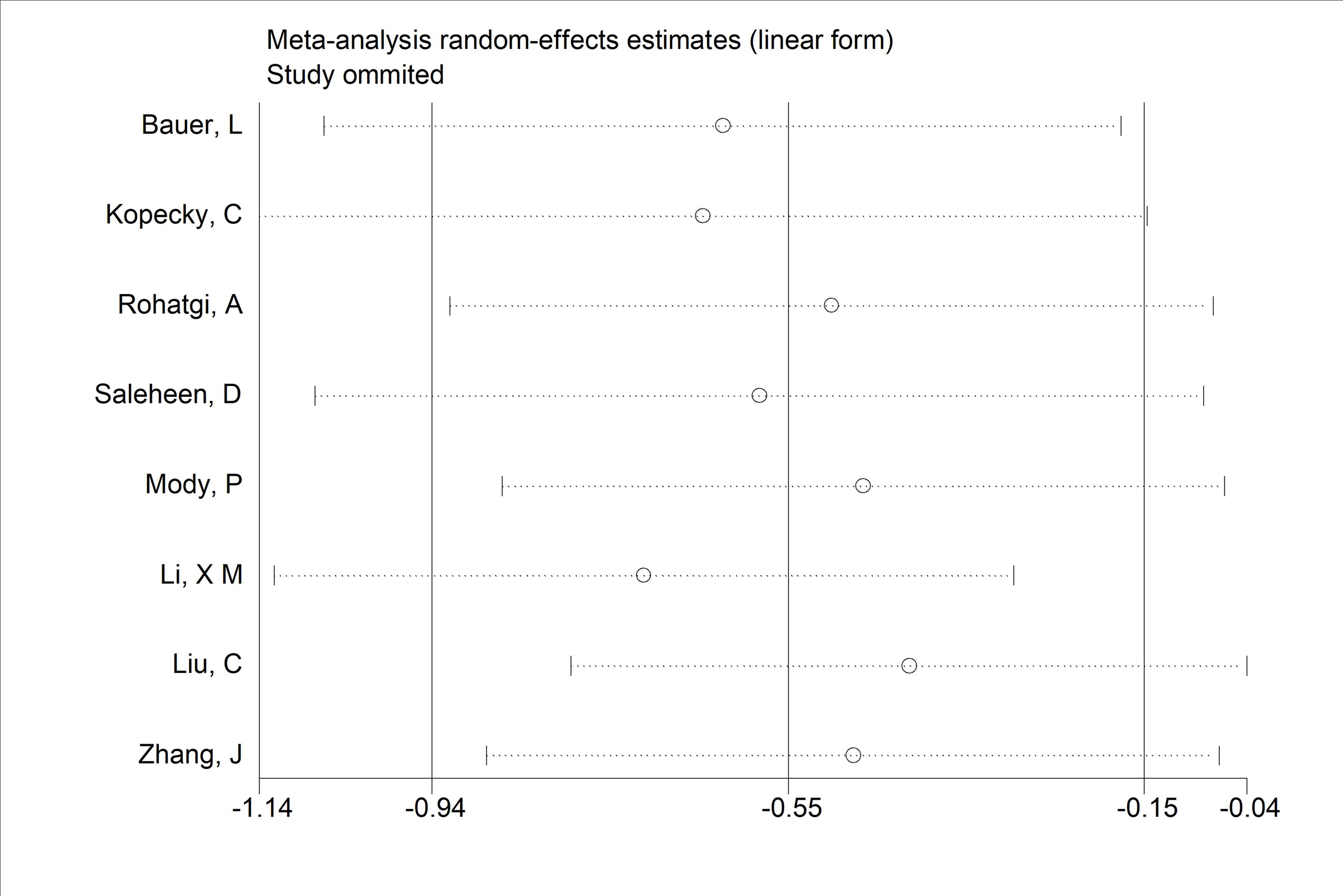

Supplement: Supplementary file 3 — Sensitivity analysis of the association between CEC and the incidence of cardiovascular events (highest vs. lowest CEC). (TIFF 9883 kb) [file 12944_2017_604_MOESM3_ESM.tif]

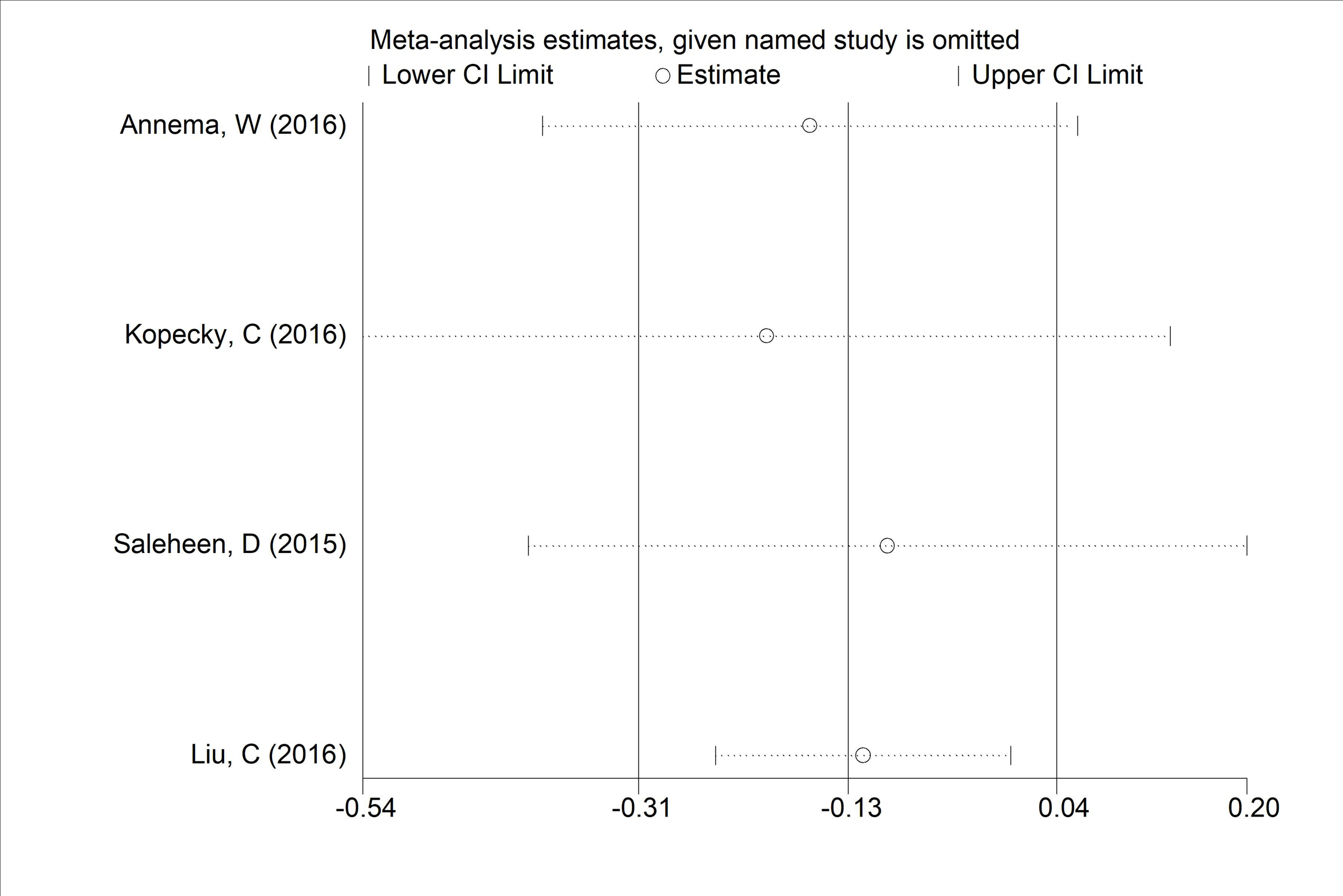

Supplement: Supplementary file 4 — Sensitivity analysis of the association between CEC and the incidence of cardiovascular events with 1 SD increase in CEC. (TIFF 9883 kb) [file 12944_2017_604_MOESM4_ESM.tif]

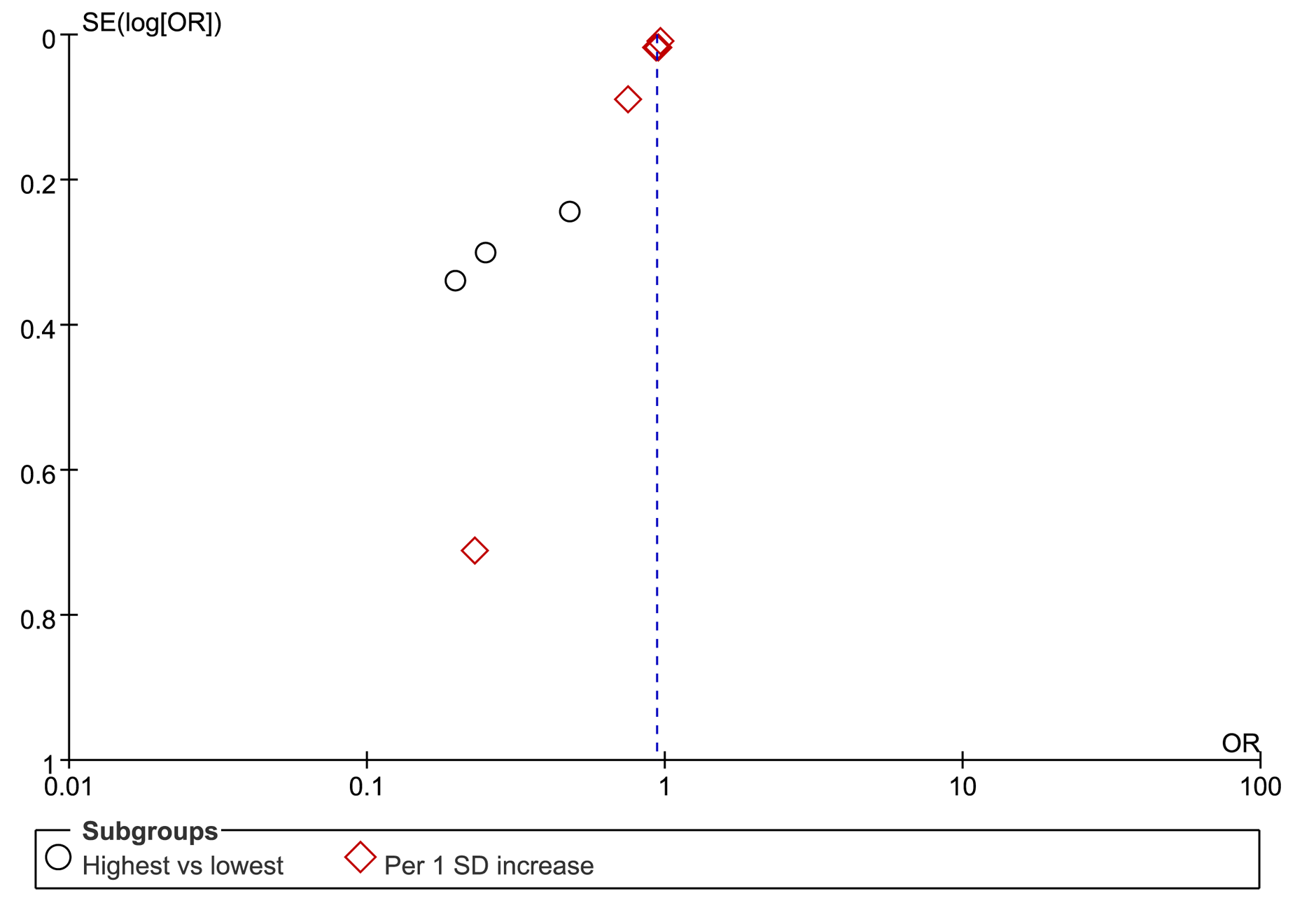

Supplement: Supplementary file 5 — Funnel plot of CEC and the prevalence of cardiovascular events. (TIFF 7496 kb) [file 12944_2017_604_MOESM5_ESM.tif]

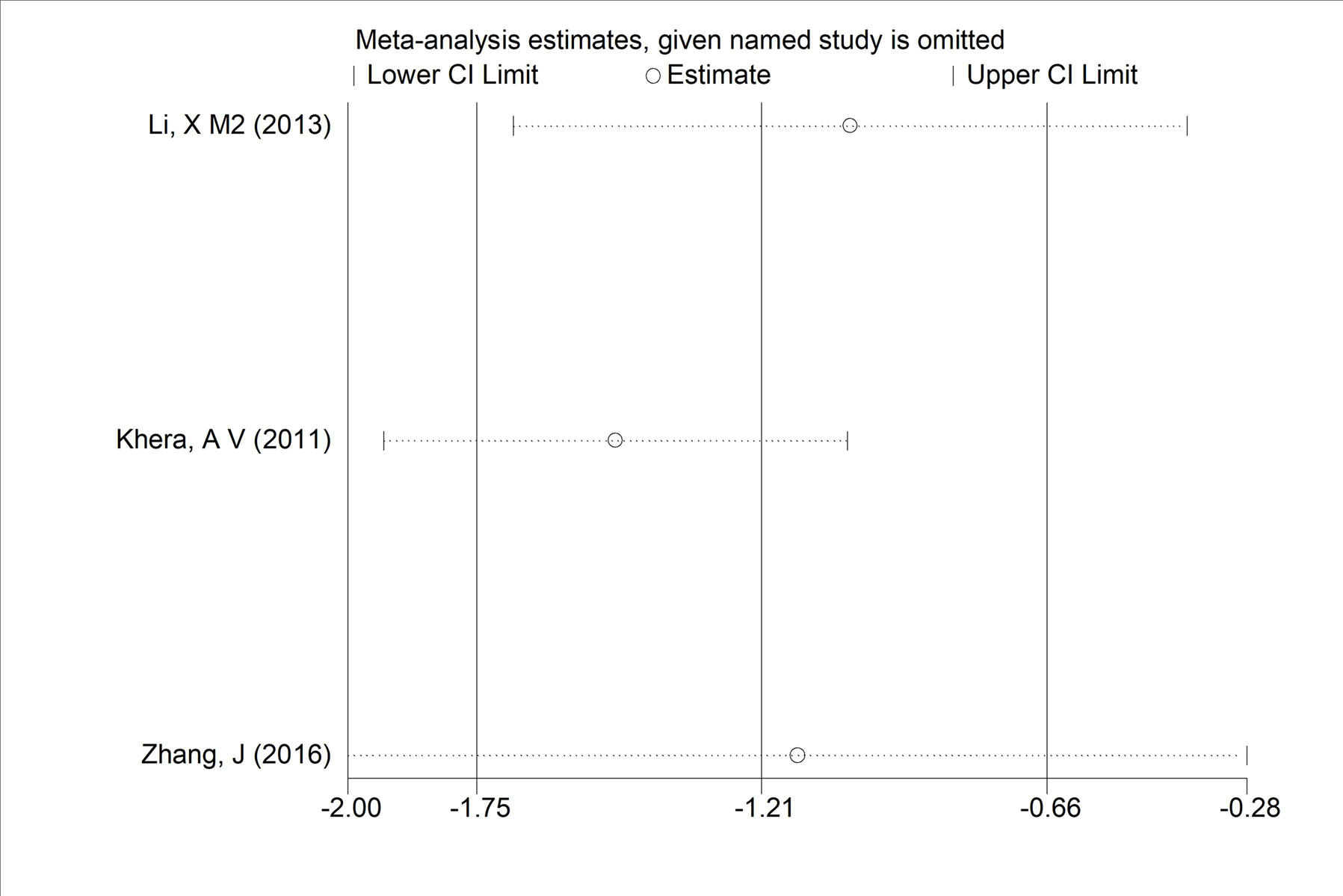

Supplement: Supplementary file 6 — Sensitivity analysis of the association between CEC and the prevalence of cardiovascular events (highest vs. lowest CEC). (TIFF 6331 kb) [file 12944_2017_604_MOESM6_ESM.tif]

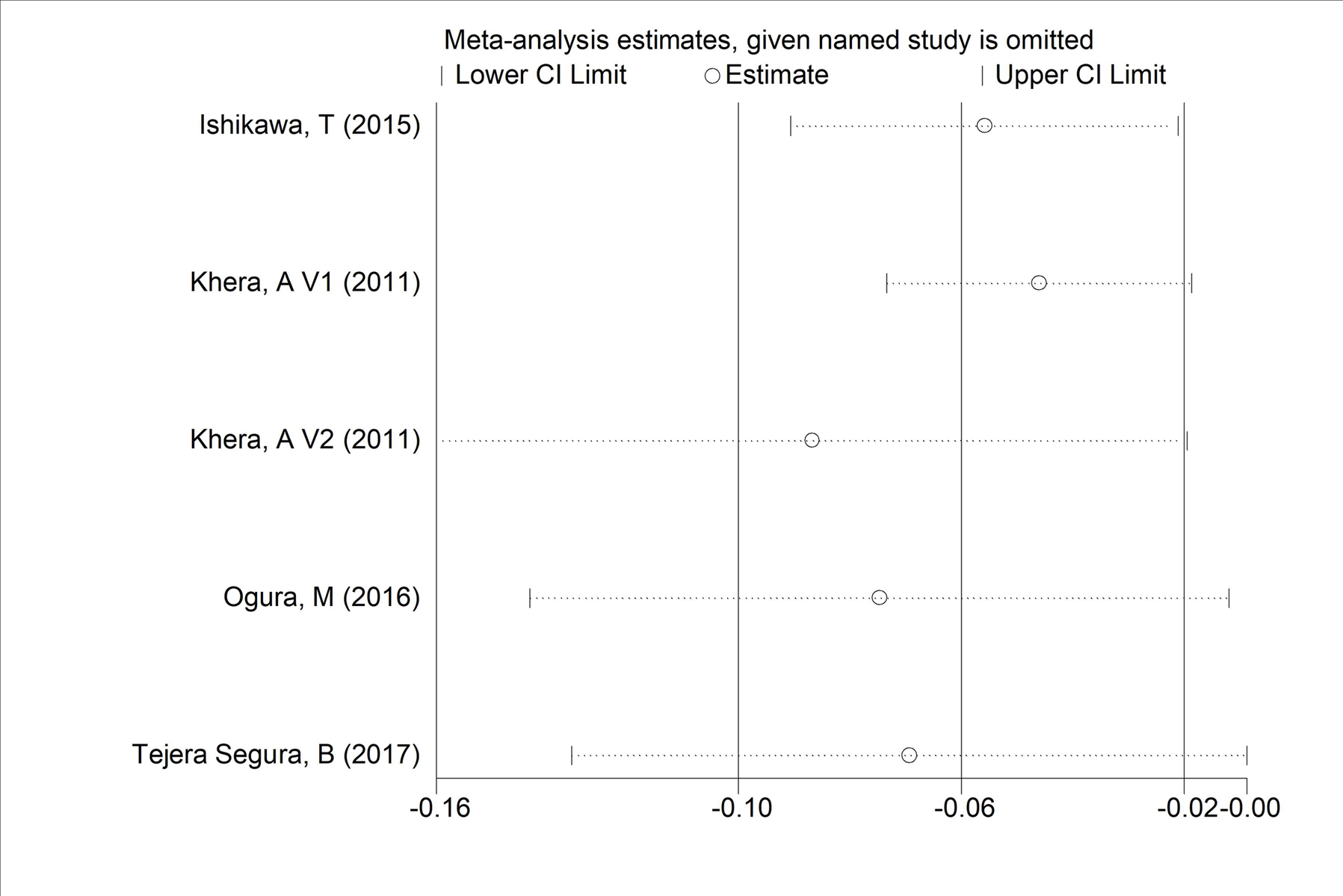

Supplement: Supplementary file 7 — Sensitivity analysis of the association between CEC and the prevalence of cardiovascular events with 1 SD increase in CEC. (TIFF 6332 kb) [file 12944_2017_604_MOESM7_ESM.tif]
